# To Explore the Active Components, Targets, and Potential Effects of Emodin in the Treatment of Colorectal Cancer Based on Network Pharmacology

**DOI:** 10.1155/ppar/6547135

**Published:** 2025-11-12

**Authors:** Libin Chen, Jiante Li, Zhicheng Cheng, Feng Jin, Jin Shi, Lifang Weng, Chunsheng He, Lijuan Wang, Zhisong Qiu

**Affiliations:** ^1^Department of Gastroenterology, Cangshan Hospital, The 900Th Hospital of Joint Logistics Support Force of Chinese People's Liberation Army, Fuzhou City, Fujian Province, China; ^2^Department of Anorectal Surgery, The Second Affiliated Hospital and Yuying Children's Hospital of Wenzhou Medical University, Wenzhou City, Zhejiang Province, China

**Keywords:** colorectal cancer, emodin, molecular docking, network pharmacology, PPAR*γ*

## Abstract

**Objective:**

The objective was to investigate the effects and potential molecular mechanisms of emodin on colorectal cancer via network pharmacology combined with experimental validation.

**Methods:**

The active components and targets of emodin were retrieved from TCMSP and BATMAN-TCM databases, while colorectal cancer (CRC)-related genes were screened via GeneCards, OMIM, and DisGeNET. The intersection targets were used to construct a compound–disease network and a protein–protein interaction (PPI) network. GO and KEGG enrichment analyses were conducted to reveal key biological functions and pathways. Molecular docking was used to assess binding affinities between core targets and active components. In vitro experiments (CCK-8, colony formation, and apoptosis assays) and in vivo xenograft models were performed to validate the antitumor effect of emodin. Quantitative real-time PCR and Western blot were used to evaluate the regulation of hub genes and signaling pathways.

**Results:**

A total of 37 active components and 235 targets of emodin were identified, of which 82 overlapped with CRC-related genes. Core targets (CASP3, MMP9, BCL2, PTGS2, and IL1B) were highlighted through network analysis. These targets were enriched in oxidative stress, apoptosis, inflammation, and metabolic pathways. Molecular docking showed strong interactions between emodin and hub targets. Emodin significantly suppressed proliferation, colony formation, and induced apoptosis in CRC cell lines in a dose-dependent manner. In vivo, emodin inhibited tumor growth and activated the PPAR*γ*–TP53 signaling axis.

**Conclusion:**

Emodin exerts anti-CRC effects via a multitarget, multipathway mechanism, particularly through modulation of the PPAR*γ*–TP53 axis. These findings support emodin's potential as a natural compound for CRC treatment.

## 1. Introduction

Colorectal cancer (CRC), a common digestive tract malignancy, sees annual increases in incidence and ranks fourth in cancer-related deaths [1]. Approximately 900,000 people die from CRC each year worldwide [2]. In our country, the improvement in economic conditions has led to an increased risk of CRC due to unfavorable risk factors such as dietary habits, lifestyle choices, obesity, and a lack of physical exercise, with a trend toward younger patients [3]. The principal treatments for CRC currently include surgical resection, chemotherapy following surgery, targeted therapy, and immunotherapy [4, 5]. Although progress in conventional chemotherapy, precision medicine, and immunotherapy has led to improved survival rates, the development of resistance to drugs and toxic side effects poses significant constraints on the application of these medical treatments in clinical settings. Therefore, finding an effective drug for treating CRC with fewer adverse reactions has become a focal point of research.

Emodin serves as the key bioactive compound in plants such as rhubarb, *Polygonum multiflorum*, and *Polygonum cuspidatum* [6]. It appears as an orange, long, needle-like crystal composed of organic substances and exhibits pharmacological, metabolic, and toxic effects. Emodin has been scientifically validated to demonstrate significant bioactivities in three therapeutic domains: oncological intervention (anticancer effects), pathophysiological regulation (anti-inflammatory and immunosuppressive actions), and physiological protection (analgesic efficacy and organoprotective properties) [7, 8]. Studies have confirmed its ability to inhibit the onset and progression of breast cancer, liver cancer, and lung cancer through various mechanisms, showcasing its strong targeting capabilities and minimal side effects [9–11]. Emodin can significantly enhance patient survival rates and reduce cancer recurrence. Additionally, it can produce synergistic effects when used in combination with other antineoplastic agents. However, its efficacy in combating CRC and the associated mechanisms of action remain inadequately established [12]. In this study, we conducted an in-depth investigation into the mechanism of emodin's action against CRC. Building upon network pharmacology and integrating transcriptomic analysis, we have for the first time unveiled the dual-axis regulatory mechanism of emodin involving peroxisome proliferator-activated receptor gamma (PPAR*γ*)–TP53, supported by corresponding in vitro and in vivo validation experiments. Through systematic network analysis, from multiple dimensions, the system-level mechanisms underlying emodin's tumor-suppressive effects. Capitalizing on recent advances in modern pharmacological approaches and interdisciplinary technological integration, this study employs network topology analysis and bioinformatics strategies to comprehensively investigate the active constituents, underlying mechanisms, and molecular targets of emodin in CRC treatment. Our multidimensional elucidation of emodin's system-level antitumor mechanisms provides a robust theoretical framework to support its clinical translation and therapeutic application against CRC.

Network pharmacology is an emerging interdisciplinary field that investigates the mechanisms of drug action and designs multitarget drug molecules from a system-level perspective [13]. With the rapid advancements in bioinformatics, systems biology, and multipharmacology, network pharmacology is increasingly recognized as a promising approach for more cost-effective drug development [14]. Network pharmacology–based approaches and molecular docking studies have gained prominence as key methodologies for identifying bioactive compounds and unraveling the underlying mechanisms of action within traditional Chinese medicine (TCM) [15]. The discovery of anticancer drugs has yielded remarkable results through the application of network pharmacological approaches.

In conclusion, this research employed an integrative network pharmacology approach to delineate molecular targets, bioactive components, and signaling pathways mediating Emodin's therapeutic efficacy against CRC. For the first time, we have combined network pharmacology with transcriptomic analysis and comprehensively validated the PPAR*γ*–TP53 dual regulatory axis mechanism through both in vitro and in vivo experiments, thereby providing a foundation for understanding the mechanisms by which emodin acts in CRC treatment and offering a more robust theoretical basis for clinical applications.

## 2. Materials and Methods

### 2.1. Network Pharmacology Analysis

#### 2.1.1. Screening of Active Components of Emodin

The active ingredients were identified by consulting the pharmacological database of the TCM system and the analysis platform TCMSP (https://old.TCMSP-e.com/TCMSP.php), as well as the Batman database (http://bionet.ncpsb.org/cnbatman-tcm). Parameters were required to meet oral bioavailability (OB) ≥ 30% and the chemical spatial distribution meets the criteria of drug − likeness (DL) ≥ 0.18, along with other default settings to filter the component information. Subsequently, the Canonical SMILES information obtained from the PubChem database was carried out via the Swiss Target Prediction platform (https://www.SwissTargetPrediction.ch/), and the screening conditions were set to be as follows: species-qualified *Homo sapiens* and binding probability threshold ≥ 0, in order to retrieve the corresponding component targets, ensuring that duplicate targets were removed.

#### 2.1.2. Acquisition of CRC Target Genes

Relevant therapeutic targets were systematically identified through multidatabase interrogation of GeneCards (https://www.genecards.org/), DisGeNET (https://www.disgenet.org/), and OMIM (https://omim.org/) platforms, employing the keyword as the principal search descriptor. As the screening criterion for the GeneCards website, choose targets with a relevance score higher than 5. To complete the CRC target list, combine these targets from the three databases and eliminate duplicates. Finally, the pharmacodisease interaction network was constructed through systematic integration of TCM bioactive constituent targets and pathological biomarkers using the Venny 2.1 visual analytics platform (http://www.bioinformatics.com.cn/static/others/jvenn/example.html), employing Venn diagrammatic overlay for target convergence analysis.

The intersection of these two sets of targets is identified as emodin, which is associated with CRC-related targets.

### 2.2. Network Construction and Hub Gene

Intersecting targets were uploaded to the STRING database (https://string-db.org) under species-specific parameters with a confidence threshold of 0.4. Cytoscape 3.8.2 was subsequently utilized to assemble and topologically analyze the PPI network, enabling identification of hub proteins and network visualization. The analysis of gene clusters and the selection of core targets were conducted using MCODE. The drug components, targets, and disease phenotypes are characterized by the nodes in the network visualization model, while their interactions are characterized by the edge connections. The network was analyzed using 10 algorithms from CytoHubba, including EPC, degree, bottleneck, radiality, and betweenness. Hub genes were identified through multialgorithm consensus, requiring genes to rank within the Top 10 positions across ≥ 5 independent computational rankings.

### 2.3. Gene Ontology (GO) Functional Enrichment and Kyoto Encyclopedia of Genes and Genomes Enrichment Analyses

GO function enrichment analysis and KEGG gene pathway enrichment for potential targets—specifically in cellular component (CC), biological process (BP), and molecular function (MF)—were conducted using the clusterProfiler package in R Version 3.5.0. The bar graph for the GO function enrichment study was made using the ggplot2 software once the Top 10 findings for BP, CC, and MF were successfully determined.

### 2.4. Molecular Docking

The primary active components were selected from the network and docked with the core target genes. Get the active components' MOL2 file from the TCMSP platform and then use ChemBio3D Ultra 14.0 to turn it into a 3D structure. The PDB database provided the core target genes' PDB format files, and the proteins were prepared using PyMOL 2.4.1 software before being docked with AutoDock Vina 1.1.2 software.

#### 2.4.1. Cell Lines

Human cell lines SW620 and HCT116 were obtained from the American Type Culture Collection (ATCC, United States). Cells were maintained in DMEM/RPMI 1640 (Gibco) containing 10% FBS under standard culture conditions (37°C, 5% CO₂) using Thermo incubators.

### 2.5. Polymerase Chain Reaction

Total RNA was extracted using RNAzol RT (GeneCopoeia, Rockville, MD, United States). The extracted RNA was reverse-transcribed using a Thermo Fisher Scientific kit to synthesize cDNA. Transcript quantification employed SYBR Green (TaKaRa) and probe-based chemistry (Roche) detection systems. Target gene expression was normalized to GAPDH and calculated via the 2^−*ΔΔ*Ct^ method. The primer sequences used in this study are summarized in Supporting Information 3: Table S1.

### 2.6. Cell Counting Kit-8 Assay

Cell viability was evaluated using the CCK-8 assay (KeyGen Biotech, Nanjing, China). Cells (1000–3000 cells) were seeded in 96-well plates, fully treated as indicated, and incubated with CCK-8 solution (10 *μ*L/mL) for 2 h. Cellular viability indices were spectrophotometrically determined at *λ* = 450 nm using a Synergy H1 multimode reader.

#### 2.6.1. Colony Formation Assays

CRC cell clonogenicity was quantified via prolonged culture (2–3 weeks) of logarithmically growing cells plated at 10^3^ cells/well under incremental compound exposure. Clonogenic efficiency, derived from (colony number)/(plated cells) × 100%, was determined after standardized fixation-staining protocol (4% PFA →0.1% crystal violet).

#### 2.6.2. Cell Apoptosis

Using phosphatidylserine externalization as an apoptotic indicator, KeyGEN's Annexin V-APC/PI detection system was implemented for flow cytometric analysis. Employing dual-parameter apoptotic detection, 5 *μ*L Annexin V-APC and 5 *μ*L PI were sequentially introduced to 500-*μ*L buffer-resuspended transfected cells. Employing BD digital cytometry technology, light-protected samples were analyzed post-15-min equilibration, with computational processing via FlowJo's advanced clustering algorithms.

#### 2.6.3. Mouse Xenograft Models

All animal procedures were approved by the Ethics Committee of the First Affiliated Hospital of Sun Yat-sen University (Approval No. [2024]086) and conducted in compliance with the relevant institutional and international guidelines. Human subcutaneous xenograft models of CRC were established using HCT116 cells [16]. Male mice were sourced from Nanjing University's animal center. Subcutaneous implantation was performed with 10^6^ cells resuspended in 100 *μ*L Matrigel-mixed PBS solution. Once the tumors reached a volume of approximately 100 mm^3^, the mice were treated with either physiological saline or experimental drugs via tail vein injection. Longitudinal tumor measurements were conducted at 3-day intervals, culminating in terminal euthanasia at the 15-day experimental endpoint. Animals were anesthetized with isoflurane gas (3%–4% for induction and 1.5%–2% for maintenance) delivered via inhalation. At the end of the experiment, euthanasia was performed using isoflurane overdose (≥ 5%), followed by cervical dislocation, in accordance with institutional ethical guidelines.

#### 2.6.4. Statistical Analysis

With three separate experimental iterations performed, all datasets were compiled from triplicate observations. Employing the nonparametric Mann–Whitney test, gene expression levels were statistically analyzed based on mean values with standard deviation measurements. Dichotomous data analysis incorporated *χ*^2^ tests with Fisher's exact alternatives where appropriate, supplemented by parametric (unpaired *t*-test and ANOVA) or nonparametric comparisons for continuous measures. Statistical analyses were conducted using SPSS 22.0 and GraphPad Prism Version 8.0. A *p* value below 0.05 was considered statistically significant.

## 3. Results

### 3.1. Screening of Active Components of *Houttuynia cordata* and Their Targets

Using the conditions of DL ≥ 0.18 and OB ≥ 30%, relevant targets for the active components of emodin were retrieved from the TCMSP database. The case of 37 active components was identified. After removing duplicates, a total of 235 unique targets were recognized.

### 3.2. Disease-Related Genes Intersect With Emodin Targets

**Figure 1:**
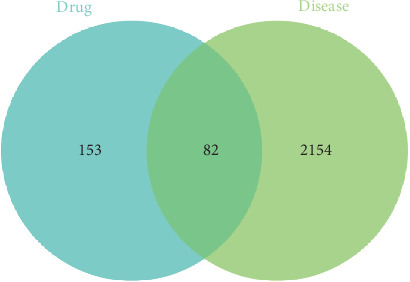
Venn diagram: 82 common targets between emodin and colorectal cancer.

Through the OMIM and GeneCards disease databases, a total of 2236 genes related to CRC were identified. An intersection of emodin targets and disease-related genes yielded 82 common genes (see Figure 1).

### 3.3. Component–Target–Disease and PPI Network Construction

**Figure 2:**
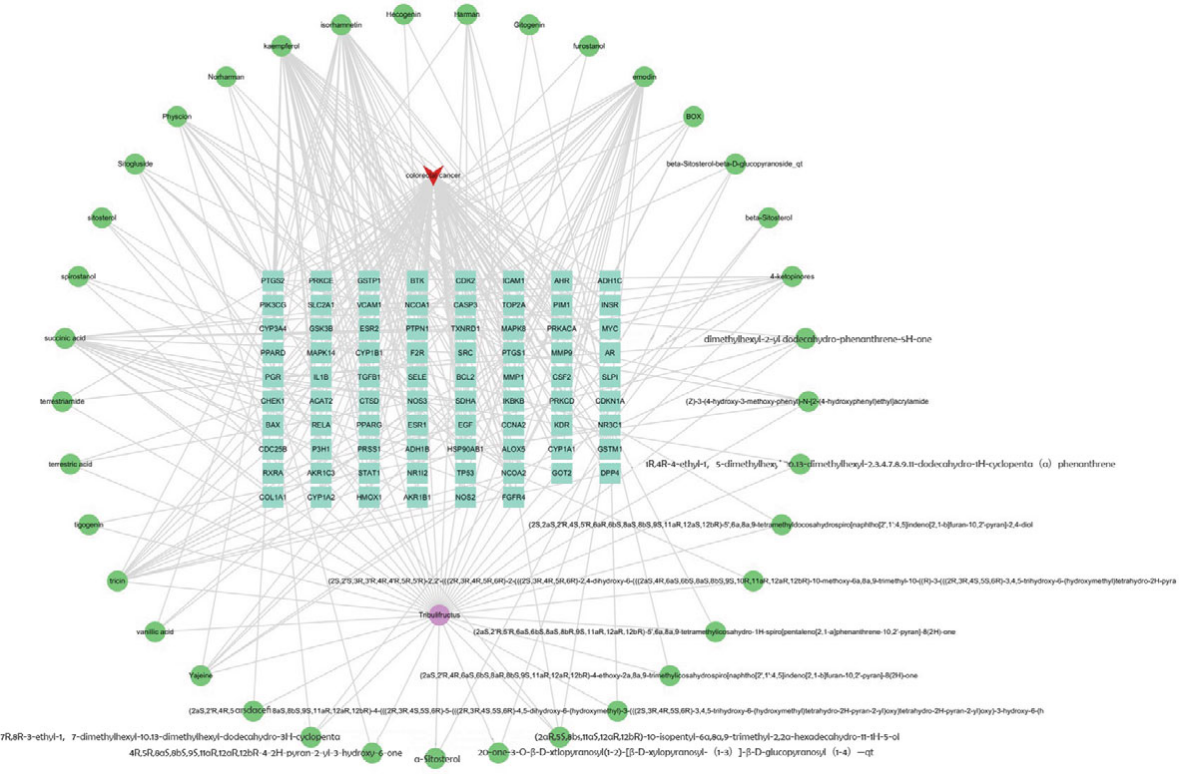
Protein–protein interaction network in colorectal cancer treatment by emodin.

**Figure 3:**
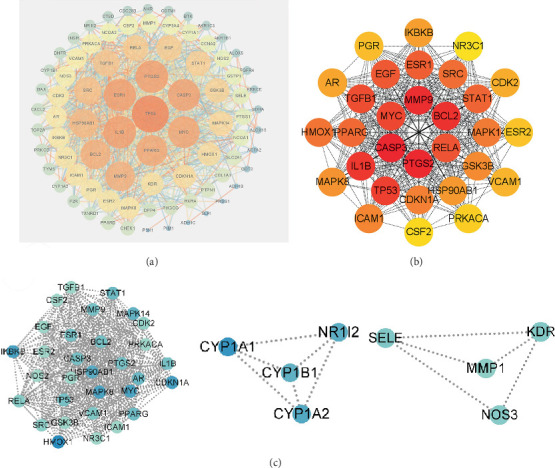
Drug-active component–target–disease and PPI network construction. (a) Emodin–colorectal cancer–target–pathway network diagram. (b) Hub-to-core target PPI network. (c) Three MCODE clustering analysis. Note: PPI, protein–protein interaction.

Then, 82 potential therapeutic targets of emodin related to CRC, along with 37 associated components, were imported into Cytoscape 3.8.0 to construct the network. The circular boxes represent the active components, the rectangular boxes represent the therapeutic targets for CRC, and the lines indicate the effects of the active components on these therapeutic targets (Figure 2). Active compounds with degrees higher than six were included, according to a degree analysis performed with the CytoNCA plug-in. Furthermore, the STRING network platform was updated to include 82 interacting targets (Figure 3a). The reason was as follows: improved clarity and readability by correcting grammatical errors, enhancing vocabulary, and ensuring technical accuracy.

To further determine the core target of emodin in CRC treatment from the 82 identified targets, the MCC algorithm was utilized within the cytoHubba plugin to select the Top 30 genes. This process resulted in the construction of a core target network, as shown in Figure 3b, comprising 30 nodes connected by edges. The intensity of the node color corresponds to the significance of each target, with darker nodes indicating higher importance. Additionally, we conducted a clustering analysis using the MCODE plugin on the PPI network in Cytoscape, resulting in three clusters (Figure 3c) that encompass 39 genes.

### 3.4. GO and KEGG Analyses

**Figure 4:**
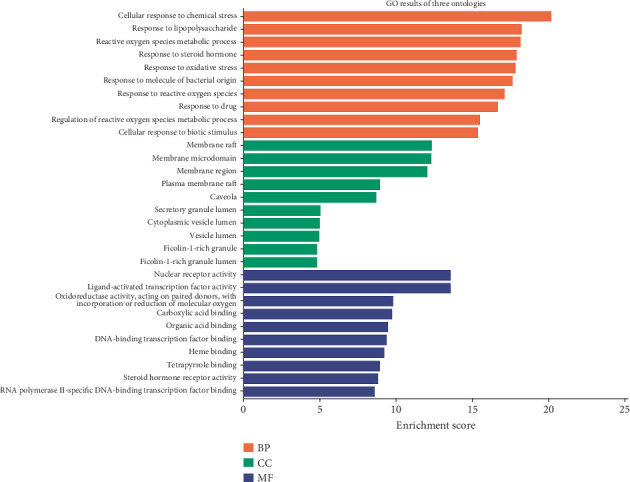
The Top 10 GO functional enrichment analyses of emodin in the treatment of colorectal cancer. Note: GO, Gene Ontology.

GO and KEGG pathway enrichment assessments were performed on 82 target genes through R programming language with the ClusterProfiler toolkit. Using a significance cutoff of *p* < 0.05, we identified statistically relevant annotations across three ontological categories: BPs, CC, and MFs. The 30 most significant GO entries from these analyses were subsequently visualized through a ranked bar graph in Figure 4. The results revealed that BPs were predominantly associated with cellular responses to chemical stress, lipopolysaccharides, oxidative stress, reactive oxygen species metabolism, steroid hormones, bacterial molecules, and epithelial cell proliferation. MFs were primarily involved in carboxylic acid binding, ligand-activated transcription factor activity, and nuclear receptor activity regulation.

**Figure 5:**
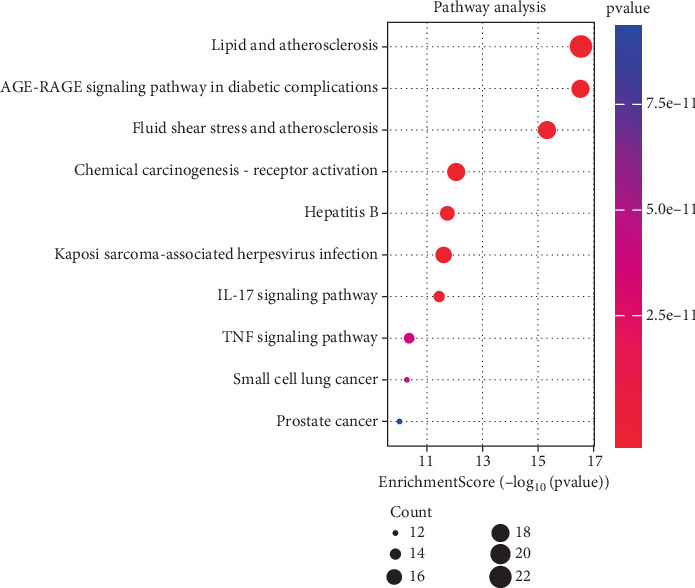
The Top 10 KEGG pathway enrichment analyses of emodin in the treatment of colorectal cancer. Note: KEGG, Kyoto Encyclopedia of Genes and Genomes.

The Top 30 significant pathways (Figure 5) were primarily involved in lipid metabolism abnormality-associated pathways, diabetic complications AGE-RAGE signaling, chemical carcinogenesis–receptor activation mechanisms, fluid shear stress–regulated atherosclerotic processes, and hepatitis B virus infection pathways, according to KEGG enrichment results based on significance threshold (*p* < 0.05) screening.

### 3.5. Molecular Docking

**Figure 6:**
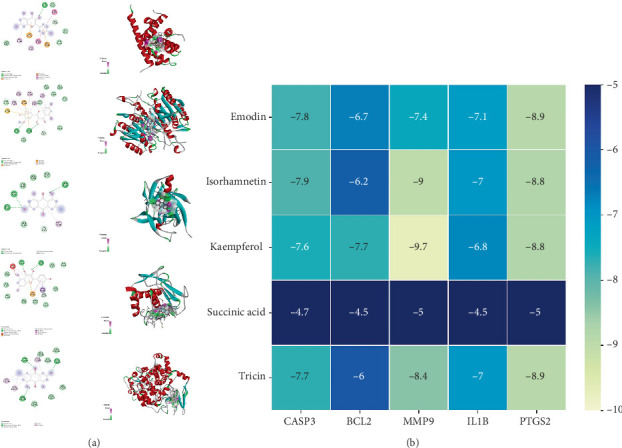
Molecular docking. (a) Molecular docking results of emodin (the docking maps of emodin with BCL-2, CASP3, il-1b, MMP, and PTGS2, respectively). (b) Heat map of docking results of emodin components with key therapeutic target molecules of colon cancer.

In the PubChem database (https://pubchem.ncbi.nlm.nih.gov/), we downloaded the 3D structures of CASP3, PTGS2, MMP9, BCL2, and IL1B to further validate the binding affinity between emodin and the identified core targets. We used the SYBYL-X 2.0 program for molecular optimization after obtaining the three-dimensional structures of the top five essential emodin components. Subsequently, we employed AutoDockTools software to perform molecular docking, using macromolecular proteins as receptors and emodin as the ligand. In the molecular docking assessment, the receptor–ligand complexes were found to exhibit binding affinities exceeding thermodynamic stability benchmarks, as evidenced by interaction energies consistently registering under − 5 kcal/mol. Such energetic profiles are indicative of viable biological binding conformations. According to established guidelines, a binding energy within − 7.0 kcal/mol signifies a strong binding interaction. The results demonstrated that kaempferol exhibited the highest binding affinity with MMP9. From a target perspective, CASP3, MMP9, and PTGS2 showed strong binding interactions with each component. From a compound perspective, both kaempferol and emodin displayed good binding affinities to various targets (Figure 6).

### 3.6. The Effects of Proliferation and Apoptosis

**Figure 7:**
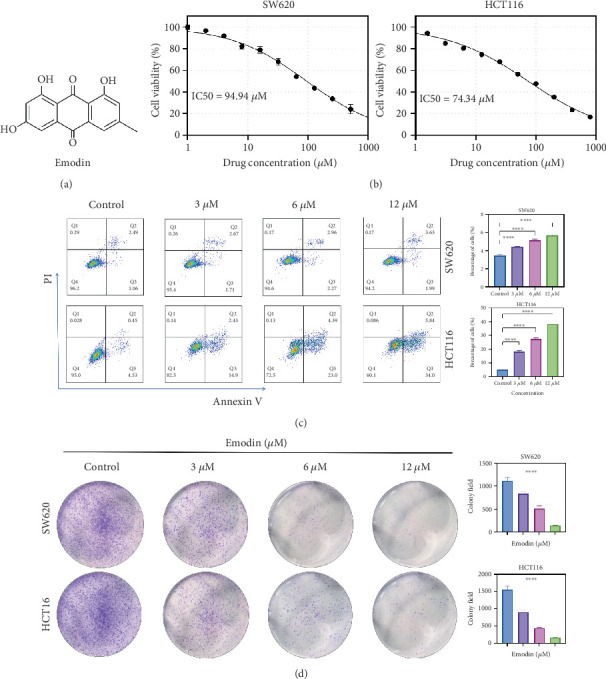
The effects of emodin on the proliferation and apoptosis of HCT116 cells in vitro. (a) The chemical structure of emodin. (b) The CCK-8 assay results measuring the activity of CRC cell lines SW620 and HCT116 treated with different doses of emodin. (c) The cell apoptosis of CRC cell lines assessed by flow cytometry. (d) The proliferation of CRC cell lines measured by the colony formation assay. All experiments were conducted in triplicate. ⁣^∗^*p* < 0.05, ⁣^∗∗^*p* < 0.01, ⁣^∗∗∗^*p* < 0.001, and ⁣^∗∗∗∗^*p* < 0.0001. Note: CRC, colorectal cancer.

Using a water-soluble tetrazolium salt-based colorimetric method (CCK-8), the cytotoxic effects of emodin on colorectal adenocarcinoma models SW620 and HCT116 were quantitatively assessed across a 6-concentration gradient (0–100 *μ*M). The inhibitory effect duration was found to be positively correlated with treatment duration, and emodin reduced the proliferation viability of both cancer cells (SW620: IC50 94.94 *μ*M and HCT116: IC50 74.23 *μ*M) in a concentration-dependent manner (Figure 7a,b). Furthermore, programmed death of SW620 and HCT116 cells was significantly higher in the emodin-treated group (*p* < 0.05), and its proapoptotic effect was positively correlated with drug concentration, as determined by a concentration gradient assay measuring apoptosis levels in CRC cells (0–120 *μ*M) (Figure 7c). Furthermore, colony formation assays were utilized to determine the influence of diverse emodin concentrations on the proliferation of SW620 and HCT116 cells. The outcome revealed that emodin suppressed CRC cell proliferation in a manner dependent on the dose (Figure 7d).

### 3.7. Regulatory Effects of Emodin on Key Genes

**Figure 8:**
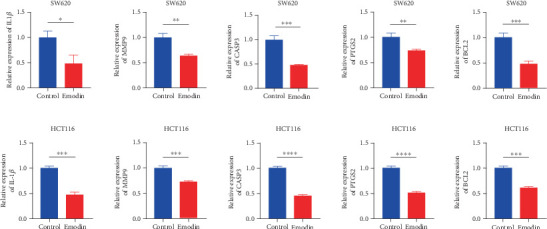
Regulatory effects of emodin on key genes. Expression levels of IL1B, MMP9, CASP3, PTGS2, and BCL2 in CRC cell lines detected by qRT-PCR. ⁣^∗^*p* < 0.05, ⁣^∗∗^*p* < 0.01, ⁣^∗∗∗^*p* < 0.001, and ⁣^∗∗∗∗^*p* < 0.0001. Note: CRC, colorectal cancer.

To investigate molecular mechanisms underlying the compound's anti-CRC effects, we applied quantitative real-time PCR (qRT-PCR) to assess key target gene expression in emodin-treated HCT116 cells, guided by core regulatory networks from network pharmacology analysis. Upon emodin administration, qRT-PCR quantification showed significant suppression of proinflammatory mediators (IL1B, MMP9, and PTGS2) and apoptotic pathway components (CASP3 and BCL2) at the mRNA abundance level in HCT116 cells versus vehicle-treated counterparts (Figure 8). By controlling important CRC genes, emodin can exert tumor suppressor effects, according to systematic predictions based on network pharmacology. Previous studies have demonstrated that PPAR-*γ* and TP53 play crucial roles in emodin-mediated regulation of CRC progression [17, 18]. Hence, we employed qPCR to examine the changes in PPAR-*γ* and TP53 expression levels in cells treated with varying concentrations of emodin. To further explore the regulatory effect of emodin on key tumor suppressor genes, we conducted qRT-PCR assays to assess mRNA expression levels of PPAR-*γ* and TP53 in HCT116 cells treated with increasing concentrations of emodin. The results showed a dose-dependent upregulation of both genes, supporting their involvement in emodin-mediated anti-CRC effects (Supporting Information 1: Figure S1).

### 3.8. Antitumor Impact of Emodin on CRC In Vivo

**Figure 9:**
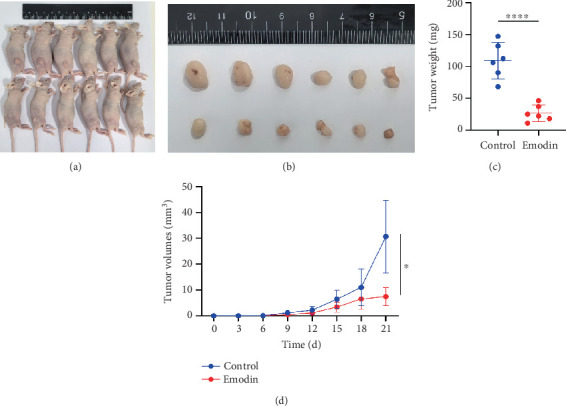
Antitumor impact of emodin on CRC in vivo. (a, b) Representative images of human CRC subcutaneous xenografted nude mouse models. (c) Tumor weight measurement. (d) Tumor volume measurement. ⁣^∗^*p* < 0.05 and ⁣^∗∗∗∗^*p* < 0.0001. Note: CRC, colorectal cancer.

Based on the results of in vitro studies, emodin has a significant impact on cells, prompting further validation of its in vivo antitumor effects. We utilized xenograft mouse models to investigate the antitumor effects of emodin on CRC (Figure 9a,b). Emodin treatment significantly decreased tumor weight and volume relative to controls (Figure 9c,d). Consistent with in vitro results, emodin-treated tumor tissues showed significantly increased expression of PPAR-*γ* and TP53 at both the mRNA and protein levels, as confirmed by qRT-PCR and Western blot assays (Supporting Information 2: Figure S2a,b). This supports the activation of the PPAR*γ*–TP53 axis in vivo as a potential mechanism for emodin's tumor-suppressive effects.

## 4. Discussion

CRC ranks third in global cancer incidence, with persistently elevated morbidity and mortality rates despite advancements in preventive and therapeutic strategies [19]. TCM, leveraging millennia of empirical applications, has emerged as a promising source for developing novel antitumor agents, particularly due to the favorable safety profiles of its bioactive constituents. Notably, emodin—a naturally derived anthraquinone—has demonstrated proapoptotic effects in breast cancer models [20], suggesting broader oncotherapeutic potential. It has been shown to lower blood lipids and improve biochemical indices and glucose metabolism in vivo [21–23]. Emodin exhibits multifaceted pharmacotherapeutic properties encompassing antineoplastic, antiallergic, bone remodeling modulation, glucose homeostasis regulation, immunomodulatory, and neuronal preservation activities. This naturally occurring anthraquinone demonstrates clinical efficacy across multiple disease domains, particularly in oncological, respiratory (asthma), degenerative joint (osteoarthritis), metabolic (diabetes mellitus), and cardiovascular (atherosclerosis) pathologies [24–26]. Emodin exerts its anti-CRC effects through the coordination and compatibility of multiple components. By constructing the drug–component network of emodin, we identified 82 potential anti-CRC targets, which are involved in various pathways. Emodin exerts its anti-CRC effects through key targets such as CASP3, PTGS2, MMP9, BCL2, and IL1B. Caspase-3, a central enzyme in apoptosis, is closely associated with processes like cardiovascular diseases, cancer, and aging. It functions as an executioner caspase activated by initiator Caspase-8 or Caspase-9, cleaving essential cellular proteins to induce apoptosis. Many anticancer therapies, including chemotherapy, radiation, and immunotherapy, trigger tumor cell death by activating Caspase-3. PTGS2, a pivotal enzyme in prostaglandin synthesis, is often induced by inflammatory stimuli and is expressed in approximately 74%–78% of CRC cases [27]. MMP-9, a well-studied matrix metalloproteinase, plays a significant role in BPs by cleaving extracellular matrix proteins and cell surface molecules, influencing ECM remodeling and various cellular functions. It is strongly implicated in cancer progression, including invasion, metastasis, and angiogenesis [28, 29]. The BCL-2 family regulates apoptosis, and its dysregulation, such as overexpression of antiapoptotic members or reduced proapoptotic proteins, is a common feature in many cancers [30]. TP53 is the most frequently mutated gene in human cancers. TP53 is a critical tumor suppressor gene that is mutated in over 50% of human cancers. These mutations not only impair its tumor-suppressive functions but also confer oncogenic properties to the mutant p53 protein [31, 32]. The p53 tumor suppressor plays a pivotal role in regulating diverse cellular processes including cell cycle control, DNA repair, apoptosis, autophagy, metabolic reprogramming, and immune responses. Given that mutant p53's oncogenic activities promote cancer proliferation and metastasis, targeting the altered signaling pathways associated with p53 mutations represents an attractive therapeutic strategy. Furthermore, literature reports indicate that emodin affects several signaling pathways, including PI3K/AKT, MAPK, NIK-IKK, MMPs, PPAR*γ*, NF-*κ*B, and activin A [33, 34]. PPAR*γ* and p53 (encoded by the TP53 gene) are two critical signaling pathways that exhibit significant cross-talk in tumorigenesis, metabolic reprograming, and cell fate determination. The interplay between these pathways forms the PPAR*γ*–p53 regulatory axis, which influences tumor progression through synergistic or antagonistic effects, particularly demonstrating important therapeutic value in CRC. PPAR*γ* directly binds to the TP53 gene promoter to upregulate p53 expression. Concurrently, ligand-activated PPAR*γ* reduces Bcl-2 expression while enhancing p53 activity, thereby inhibiting tumor cell cycle progression, proliferation, and in vitro invasive capacity. In contrast, mutant p53 variants (e.g., R175H and R273H) can bind to the PPAR*γ* promoter to suppress its transcription, resulting in metabolic dysregulation (e.g., enhanced glycolysis) and deterioration of the inflammatory tumor microenvironment. In summary, PPAR*γ* exerts antitumor effects by modulating multiple oncogenic pathways, including cellular differentiation, proliferation, apoptosis, inflammation and angiogenesis. The natural compound emodin demonstrates significant therapeutic potential due to its multitarget properties, particularly its dual regulatory effects on both PPAR*γ* and p53 pathways, thereby supporting its traditional use in cancer therapy.

Network pharmacology–based predictions and in vitro functional experiments jointly demonstrated the mechanism by which emodin treats CRC. The CCK-8 assay indicated that emodin significantly suppressed proliferative vitality of HCT116 and SW620 cells, with the inhibitory effect positively correlating with drug concentration (IC50 = 82.3 ± 4.1* μ*M and 67.5 ± 3.8* μ*M). Apoptosis, a key process in cancer treatment, was notably induced by emodin in both HCT116 and SW620 cells, as confirmed by flow cytometry assays. This indicates emodin's potential to trigger apoptosis in CRC cells. Colony formation assays further confirmed emodin's suppressive effects on the proliferative ability of HCT116 and SW620 cells. Most importantly, emodin significantly inhibited the growth of xenograft tumors in nude mice, highlighting its potential efficacy in CRC treatment. However, this article has some significant drawbacks. We found that relying on a large number of internet databases for CRC analysis was insufficient, and more reliable data sources should be incorporated to enhance the rigor of this study.

## 5. Conclusion

In summary, emodin exhibits antitumor effects, primarily by inhibiting the proliferation of tumor cells and affecting the progression of the tumor cell cycle. It demonstrates broad-spectrum antitumor activity, effectively suppressing the growth of cancer cells. The mechanisms through which emodin operates include inhibiting cell proliferation, inducing apoptosis, and playing a crucial role in cell cycle arrest. This paper employs network pharmacology in conjunction with molecular docking technology to study the effects of emodin on colon cancer, providing reliable information to support subsequent treatment strategies.

## Data Availability

The data underlying this article are available in the article and in its supporting information. The datasets are available via The Cancer Genome Atlas (TCGA) (https://portal.gdc.cancer.gov) and the Genotype Tissue Expression (GTEx) portal (https://xenabrowser.net/).

## Supporting Information

Supporting Information 1 Figure S1: The impact of Emodin on PPAR-*γ* and P53 expression in CRC cells was evaluated by qRT-PCR across a range of concentrations.

Supporting Information 2 Figure S2: The expression levels of PPAR and P53 in the control and emodin-treated groups were measured by Western blot (WB) (a) and qRT-PCR (b) in vivo.

Supporting Information 3 Table S1: Primer sequences for qRT-PCR.

## References

[1] Dekker E., Tanis P. J., Vleugels J. L. A., Kasi P. M., Wallace M. B. (2019). Colorectal Cancer. *Lancet*.

[2] Shin A. E., Giancotti F. G., Rustgi A. K. (2023). Metastatic Colorectal Cancer: Mechanisms and Emerging Therapeutics. *Trends in Pharmacological Sciences*.

[3] Biller L. H., Schrag D. (2021). Diagnosis and Treatment of Metastatic Colorectal Cancer: A Review. *Jama*.

[4] Li N., Lu B., Luo C. (2021). Incidence, Mortality, Survival, Risk Factor and Screening of Colorectal Cancer: A Comparison Among China, Europe, and Northern America. *Cancer Letters*.

[5] Abedizadeh R., Majidi F., Khorasani H. R., Abedi H., Sabour D. (2024). Colorectal Cancer: A Comprehensive Review of Carcinogenesis, Diagnosis, and Novel Strategies for Classified Treatments. *Cancer and Metastasis Reviews*.

[6] Dong X., Fu J., Yin X. (2016). Emodin: A Review of Its Pharmacology, Toxicity and Pharmacokinetics. *Phytotherapy Research*.

[7] Luo N., Fang J., Wei L. (2021). Emodin in Atherosclerosis Prevention: Pharmacological Actions and Therapeutic Potential. *European Journal of Pharmacology*.

[8] Dai S., Chen Y., Fan X. (2023). Emodin Attenuates Cardiomyocyte Pyroptosis in Doxorubicin-Induced Cardiotoxicity by Directly Binding to GSDMD. *Phytomedicine*.

[9] Zou G., Zhang X., Wang L. (2020). Herb-Sourced Emodin Inhibits Angiogenesis of Breast Cancer by Targeting VEGFA Transcription. *Theranostics*.

[10] Zhu M., He Q., Wang Y. (2023). Exploring the Mechanism of Aloe-Emodin in the Treatment of Liver Cancer Through Network Pharmacology and Cell Experiments. *Frontiers in Pharmacology*.

[11] Zhang F. Y., Li R. Z., Xu C. (2022). Emodin Induces Apoptosis and Suppresses Non-Small-Cell Lung Cancer Growth via Downregulation of sPLA2-IIa. *Phytomedicine*.

[12] Shen Z., Zhao L., Yoo S. A. (2024). Emodin Induces Ferroptosis in Colorectal Cancer Through NCOA4-Mediated Ferritinophagy and NF-*κ*b Pathway Inactivation. *Apoptosis*.

[13] Shang L., Wang Y., Li J. (2023). Mechanism of Sijunzi Decoction in the Treatment of Colorectal Cancer Based on Network Pharmacology and Experimental Validation. *Journal of Ethnopharmacology*.

[14] Nogales C., Mamdouh Z. M., List M., Kiel C., Casas A. I., Schmidt H. H. H. W. (2022). Network Pharmacology: Curing Causal Mechanisms Instead Of Treating Symptoms. *Trends in Pharmacological Sciences*.

[15] Sun L., Xu Y., Chen N. (2024). Chinese Herbal Medicine (JianPi-BuShen) and Completion Rate of Adjuvant Chemotherapy for Patients With Stage II and III Colon Cancer: A Randomized Clinical Trial. *European Journal of Cancer*.

[16] Tang Q., Chen J., Di Z. (2020). TM4SF1 Promotes EMT and Cancer Stemness via the Wnt/*β*-Catenin/SOX2 Pathway in Colorectal Cancer. *Journal of Experimental & Clinical Cancer Research*.

[17] Shrimali D., Shanmugam M. K., Kumar A. P. (2013). Targeted Abrogation of Diverse Signal Transduction Cascades by Emodin for the Treatment of Inflammatory Disorders and Cancer. *Cancer Letters*.

[18] Murley J. S., Arbiser J. L., Weichselbaum R. R., Grdina D. J. (2018). ROS Modifiers and NOX4 Affect the Expression of the Survivin-Associated Radio-Adaptive Response. *Free Radical Biology and Medicine*.

[19] Klimeck L., Heisser T., Hoffmeister M., Brenner H. (2023). Colorectal Cancer: A Health and Economic Problem. *Best Practice & Research Clinical Gastroenterology*.

[20] Chen Q., Tian S., Zhu J. (2016). Exploring a Novel Target Treatment on Breast Cancer: Aloe-Emodin Mediated Photodynamic Therapy Induced Cell Apoptosis and Inhibited Cell Metastasis. *Anti-Cancer Agents in Medicinal Chemistry*.

[21] Cheng L., Zhang S., Shang F. (2021). Emodin Improves Glucose and Lipid Metabolism Disorders in Obese Mice *via* Activating Brown Adipose Tissue and Inducing Browning of White Adipose Tissue. *Frontiers in Endocrinology*.

[22] Hu Z., Cheng X., Cai J., Huang C., Hu J., Liu J. (2024). Emodin Alleviates Cholestatic Liver Injury by Modulating Sirt 1/Fxr Signaling Pathways. *Scientific Reports*.

[23] Wang Y., Zhang J., Xu Z. (2022). Identification and Action Mechanism of Lipid Regulating Components From Rhei Radix et Rhizoma. *Journal of Ethnopharmacology*.

[24] Dong X., Zeng Y., Liu Y. (2020). Aloe-Emodin: A Review of Its Pharmacology, Toxicity, and Pharmacokinetics. *Phytotherapy Research*.

[25] Zheng Q., Li S., Li X., Liu R. (2021). Advances in the Study of Emodin: An Update on Pharmacological Properties and Mechanistic Basis. *Chinese Medicine*.

[26] Semwal R. B., Semwal D. K., Combrinck S., Viljoen A. (2021). Emodin - A Natural Anthraquinone Derivative With Diverse Pharmacological Activities. *Phytochemistry*.

[27] Venè R., Costa D., Augugliaro R. (2020). Evaluation of Glycosylated PTGS2 in Colorectal Cancer for NSAIDS-Based Adjuvant Therapy. *Cells*.

[28] Chang M., Nguyen T. T. (2021). Strategy for Treatment of Infected Diabetic Foot Ulcers. *Accounts of Chemical Research*.

[29] Tóth E., Turiák L., Visnovitz T. (2021). Formation of a Protein Corona on the Surface of Extracellular Vesicles in Blood Plasma. *Journal of Extracellular Vesicles*.

[30] Merino D., Lok S. W., Visvader J. E., Lindeman G. J. (2016). Targeting BCL-2 to Enhance Vulnerability to Therapy in Estrogen Receptor- Positive Breast Cancer. *Oncogene*.

[31] Hu J., Cao J., Topatana W. (2021). Targeting Mutant p53 for Cancer Therapy: Direct and Indirect Strategies. *Journal of Hematology & Oncology*.

[32] Tornesello M. L. (2024). TP53 Mutations in Cancer: Molecular Features and Therapeutic Opportunities (Review). *International Journal of Molecular Medicine*.

[33] Huang W., Zhou P., Zou X., Liu Y., Zhou L., Zhang Y. (2024). Emodin Ameliorates Myocardial Fibrosis in Mice by Inactivating the ROS/PI3K/Akt/mTOR Axis. *Clinical and Experimental Hypertension*.

[34] Gao J., Tao J., Zhang N. (2015). Formula Optimization of the Jiashitang Scar Removal Ointment and Antiinflammatory Compounds Screening by NF-*κ*B Bioactivity-Guided Dual-Luciferase Reporter Assay System. *Phytotherapy Research*.

