# Supplementary figures and images for: To Explore the Active Components, Targets, and Potential Effects of Emodin in the Treatment of Colorectal Cancer Based on Network Pharmacology
Source: PPAR Res. 2025 Nov 12;2025:6547135. doi: 10.1155/ppar/6547135 (PMC12629705; doi:10.1155/ppar/6547135)

HCT116

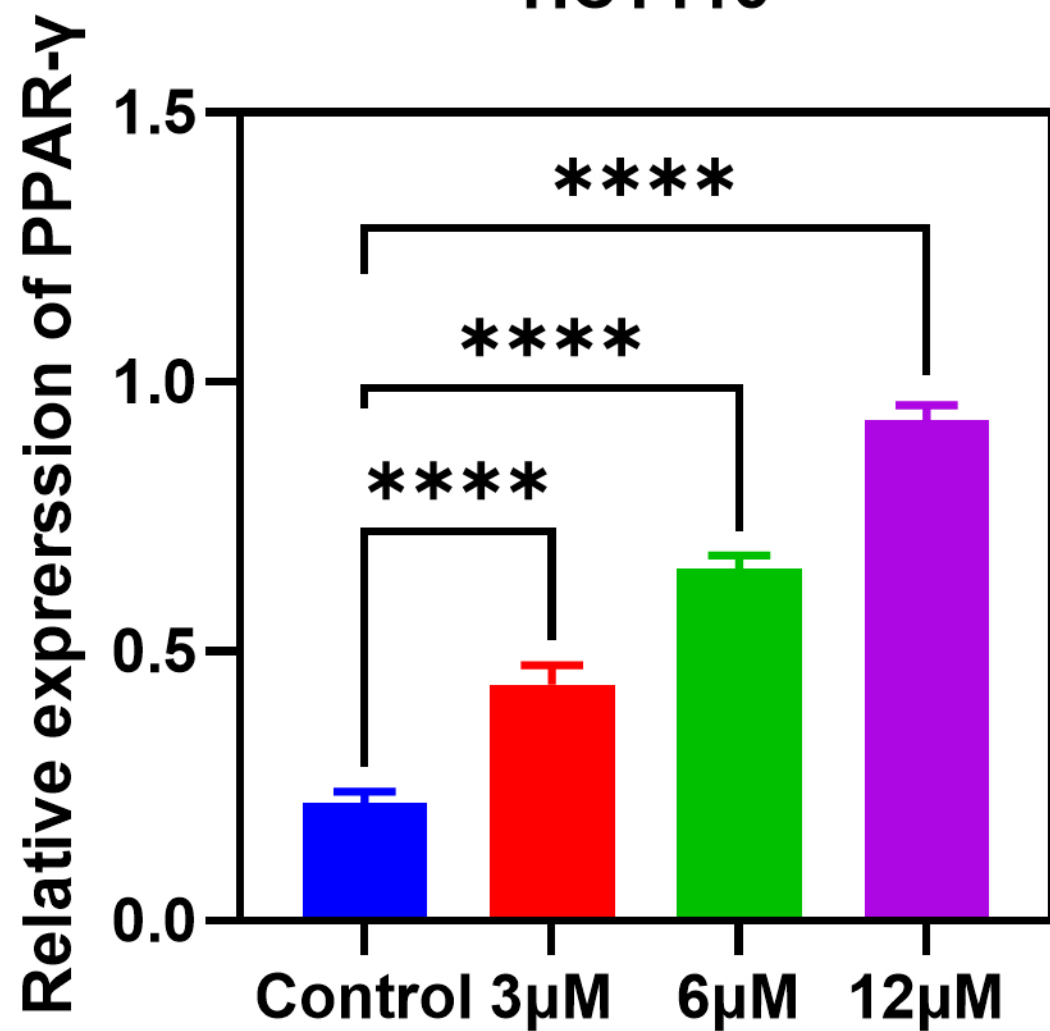

HCT116

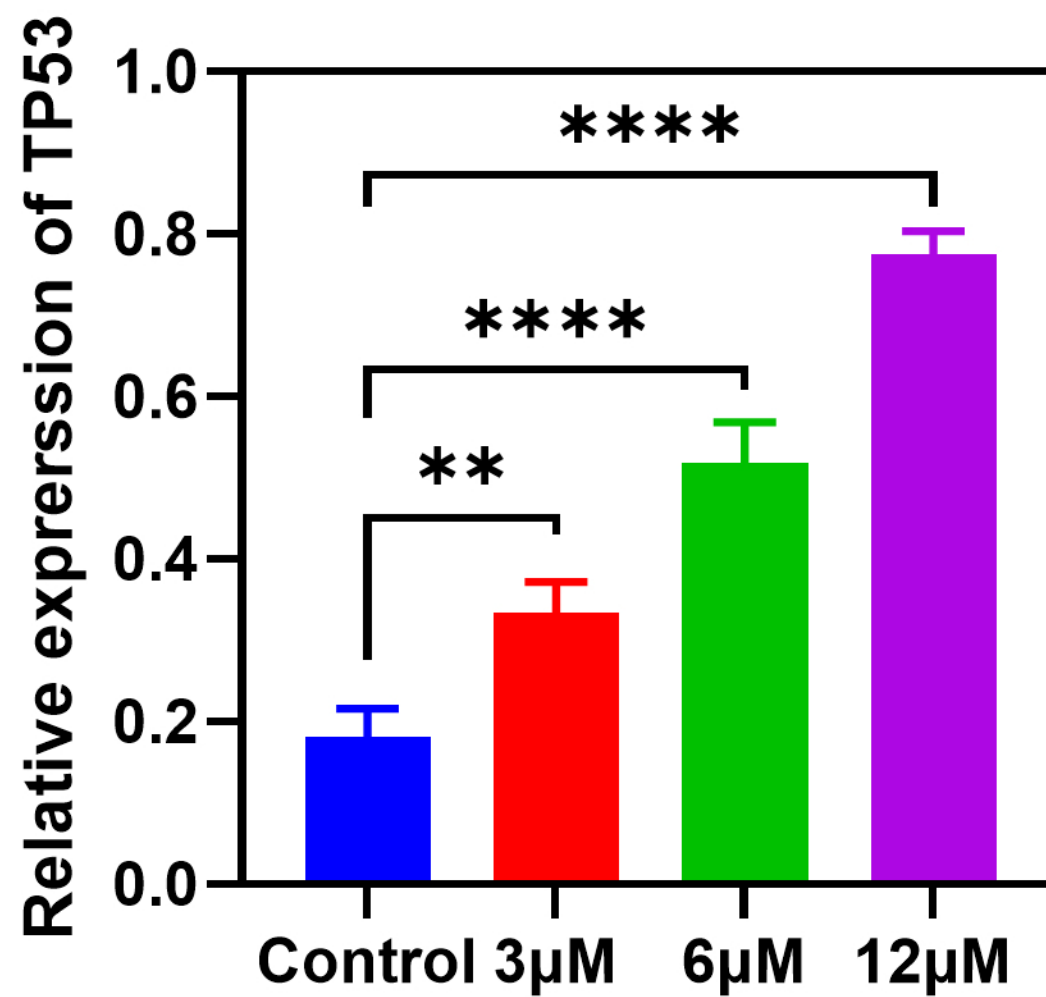

SW620

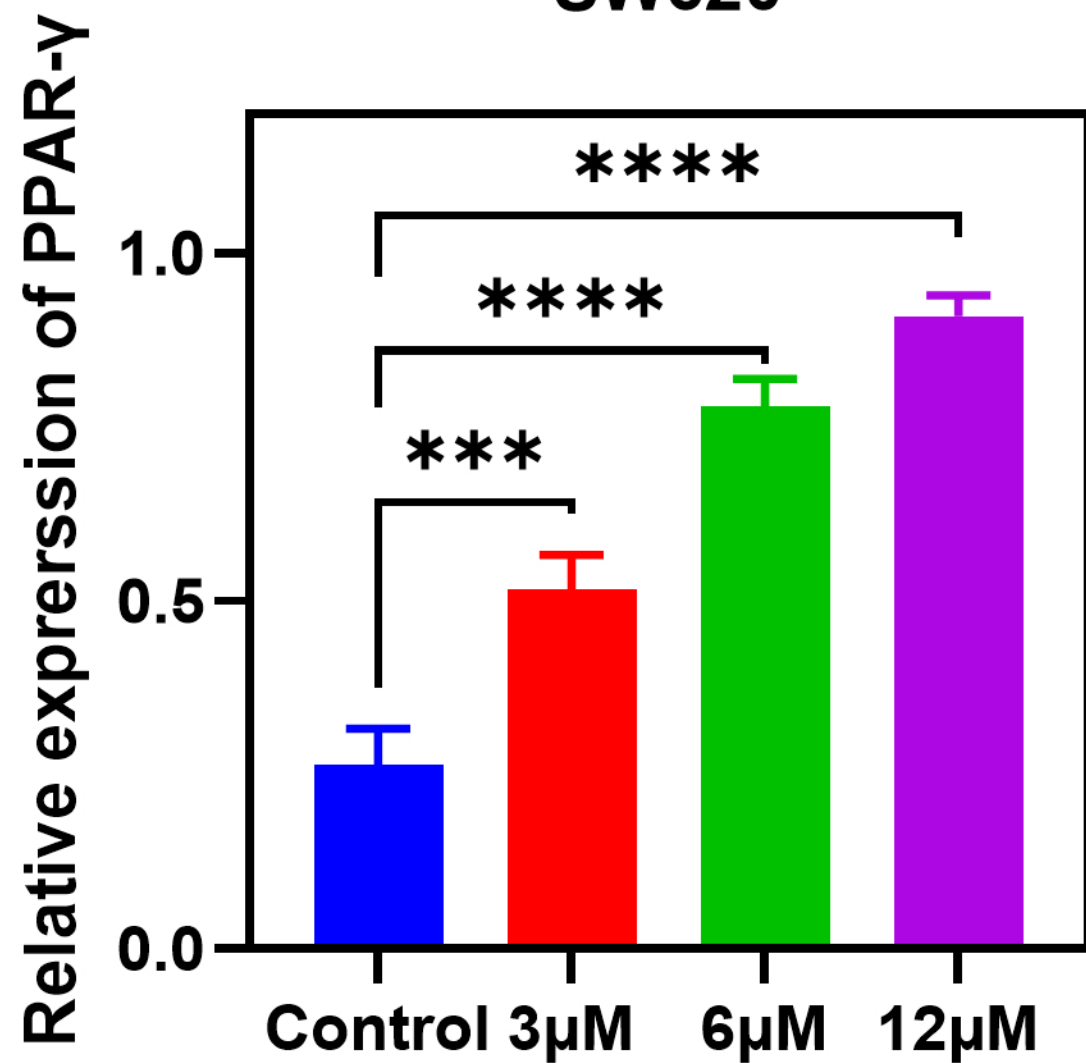

SW620

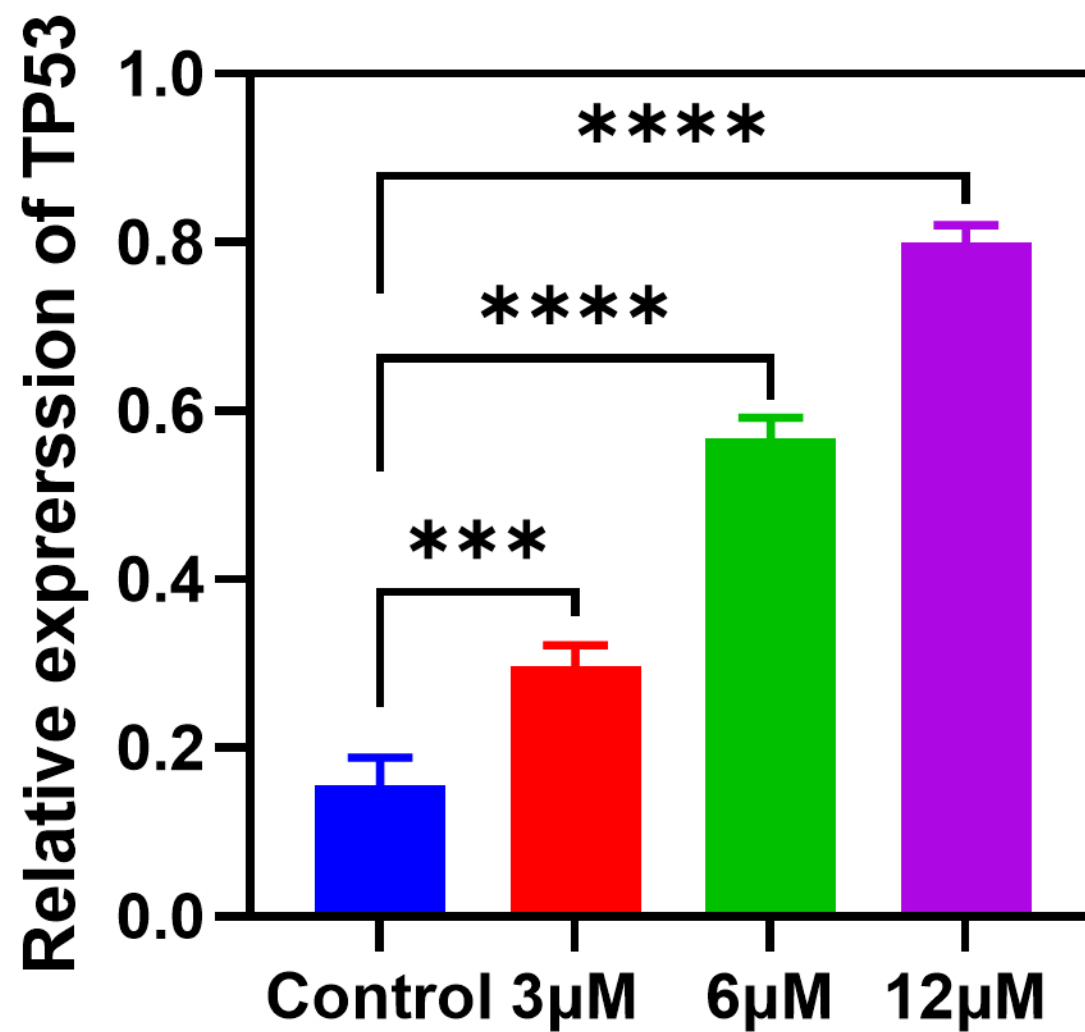

Supplement: Supporting Information 1 — Figure S1: The impact of Emodin on PPAR-γ and P53 expression in CRC cells was evaluated by qRT-PCR across a range of concentrations. [file 6547135.f1.pdf]
