# Supplementary material for: To Explore the Active Components, Targets, and Potential Effects of Emodin in the Treatment of Colorectal Cancer Based on Network Pharmacology
Source: PPAR Res. 2025 Nov 12;2025:6547135. doi: 10.1155/ppar/6547135 (PMC12629705; doi:10.1155/ppar/6547135)
Supplement: Supporting Information 2 — Figure S2: The expression levels of PPAR and P53 in the control and emodin-treated groups were measured by Western blot (WB) (a) and qRT-PCR (b) in vivo. [file 6547135.f2.pdf]

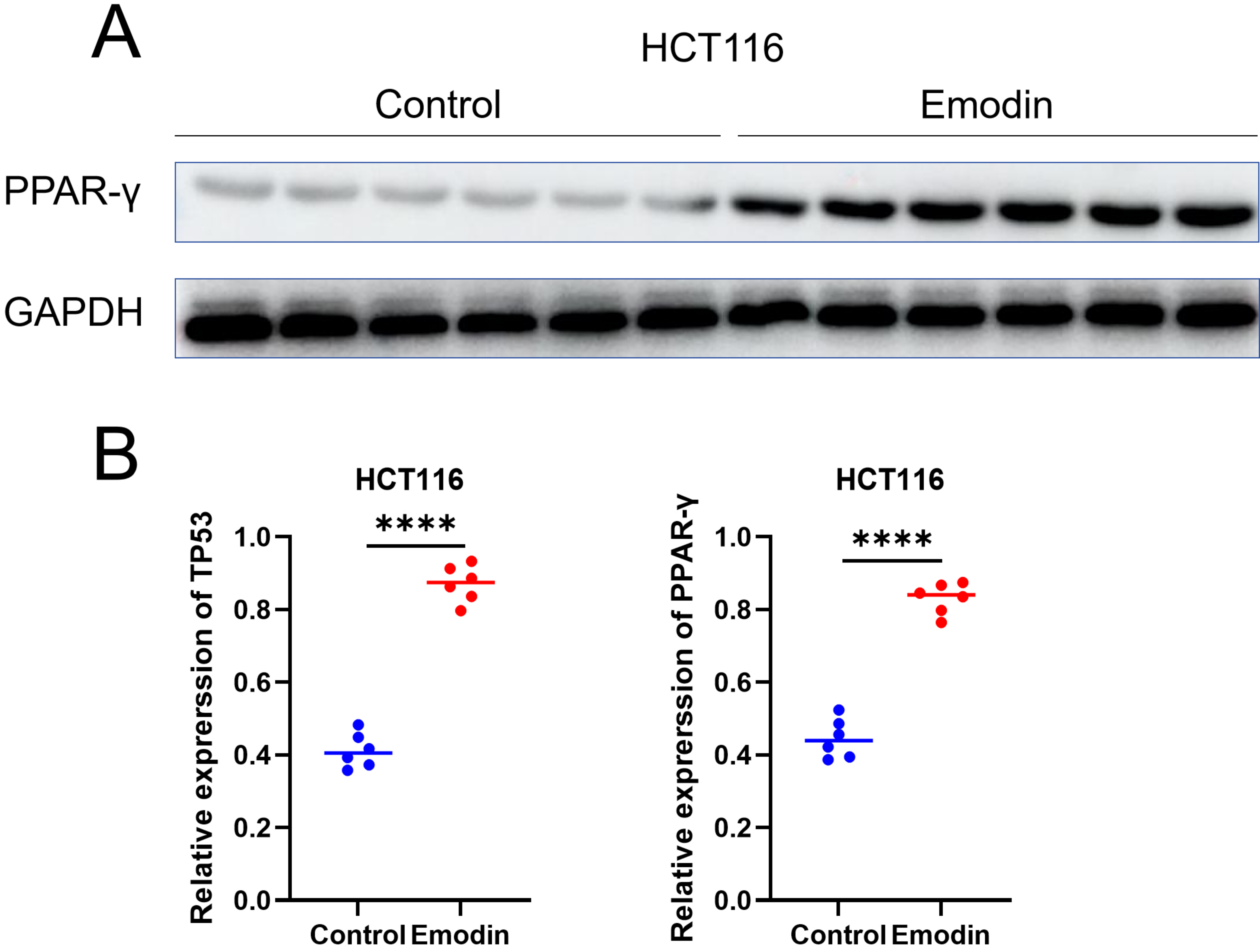

A
HCT116
Control
Emodin
PPAR-γ
GAPDH
B
HCT116
Relative expression of TP53
****
1.0
0.8
0.6
0.4
0.2
0.0
Control
Emodin
HCT116
Relative expression of PPAR-γ
****
1.0
0.8
0.6
0.4
0.2
0.0
Control
Emodin
