# Supplementary material for: To Explore the Active Components, Targets, and Potential Effects of Emodin in the Treatment of Colorectal Cancer Based on Network Pharmacology
Source: PPAR Res. 2025 Nov 12;2025:6547135. doi: 10.1155/ppar/6547135 (PMC12629705; doi:10.1155/ppar/6547135)
Supplement: Supporting Information 3 — Table S1: Primer sequences for qRT-PCR. [file 6547135.f3.docx]

| Table 1. Primer sequences for qRT-PCR | |
| --- | --- |
| Primers | Sequence (5′-3′) |
| CASP3 |  |
| Forwad | CATGGAAGCGAATCAATGGACT |
| Reverse | CTGTACCAGACCGAGATGTCA |
| MMP9 |  |
| Forwad | TGTACCGCTATGGTTACACTCG |
| Reverse | GGCAGGGACAGTTGCTTCT |
| IL-1β |  |
| Forwad | ATGATGGCTTATTACAGTGGCAA |
| Reverse | GTCGGAGATTCGTAGCTGGA |
| PTGS2 |  |
| Forwad | CTGGCGCTCAGCCATACAG |
| Reverse | CGCACTTATACTGGTCAAATCCC |
| GAPDH |  |
| Forwad | CACCGTCAAGGCTGAGAACG |
| Reverse | GCCCCACTTGATTTTGGAGG |
